# Dual Nanostructured Lipid Carriers/Hydrogel System for Delivery of Curcumin for Topical Skin Applications

**DOI:** 10.3390/biom12060780

**Published:** 2022-06-03

**Authors:** Rosa Calderon-Jacinto, Pietro Matricardi, Virginie Gueguen, Graciela Pavon-Djavid, Emmanuel Pauthe, Violeta Rodriguez-Ruiz

**Affiliations:** 1 ERRMECe Laboratory, Biomaterials for Health Group, CY Cergy Paris Université, Maison Internationale de la Recherche, I MAT, 1 rue Descartes, 95031 Neuville sur Oise, France; rosa.calderon-jacinto@cyu.fr (R.C.-J.); emmanuel.pauthe@cyu.fr (E.P.); 2 Department of Drug Chemistry and Technologies, Sapienza University of Rome, P.le Aldo Moro 5, 00185 Rome, Italy; pietro.matricardi@uniroma1.it; 3 INSERM U1148, Laboratory for Vascular Translational Science, Cardiovascular Bioengineering, Université Sorbonne Paris Nord, 99 Av. Jean-Baptiste Clément, 93430 Villetaneuse, France; virginie.gueguen@univ-paris13.fr (V.G.); graciela.pavon@univ-paris13.fr (G.P.-D.)

**Keywords:** nanostructured lipid carriers, curcumin, hydrogel, oxidative stress, skin applications, topical, drug delivery, fibroblasts, keratinocytes, antioxidants

## Abstract

This work focuses on the development and evaluation of a dual nanostructured lipid carrier (NLC)/Carbopol^®^-based hydrogel system as a potential transporter for the topical delivery of curcumin to the skin. Two populations of different sized negatively charged NLCs (P1, 70–90 nm and P2, 300–350 nm) were prepared and characterized by means of dynamic light scattering. NLCs presented an ovoid platelet shape confirmed by transmission electron microscopy techniques. Curcumin NLC entrapment efficiency and release profiles were assessed by HPLC (high pressure liquid chromatography) and spectrophotometric methods. Preservation and enhancement of curcumin (CUR) antioxidant activity in NLCs (up to 7-fold) was established and cell viability assays on fibroblasts and keratinocytes indicated that CUR-NLCs are non-cytotoxic for concentrations up to 10 μM and exhibited a moderate anti-migration/proliferation effect (20% gap reduction). CUR-NLCs were then embedded in a Carbopol^®^-based hydrogel without disturbing the mechanical properties of the gel. Penetration studies on Franz diffusion cells over 24 h in CUR-NLCs and CUR-NLCs/gels demonstrated an accumulation of CUR in Strat-M^®^ membranes of 22% and 5%, respectively. All presented data support the use of this new dual CUR-NLC/hydrogel system as a promising candidate for adjuvant treatment in topical dermal applications.

## 1. Introduction

Skin diseases are classified as the fourth leading cause of non-fatal morbidity worldwide and are recognized to disturb the well-being of patients, having, in some cases, consequences for the patient’s mental health and social life [1,2]. Some of these dermatological disorders, such as psoriasis [3] or atopic dermatitis [4], are directly related to chronic inflammatory processes and to prooxidant–antioxidant imbalance leading to oxidative stress.

Natural bioactive compounds (NBC) presenting anti-inflammatory and antioxidant properties, can contribute to stopping inflammation and restoring the redox balance necessary to reestablish normal dermal conditions [5]. In this regard, the topical use of NBC such as astaxanthin [6], gallic acid [7] or curcumin [8], has been described for the treatment of some skin pathologies.

Dermal topical therapy is a simple and non-invasive route of administration, which allows local delivery of high amounts of active ingredient at the site of action, improves compliance and limits some of the side effects related with systemic routes [9]. NBC are usually administered as adjuvant therapies to conventional treatments and their natural origin is usually associated with a low toxicity and a high biological activity [10]. In particular, the use of curcumin as adjuvant therapy for the topical treatment of psoriasis [11,12] has been reported. Curcumin (1,7-bis-(4-hydroxy-3-methoxyphenyl)-hepta-1,6-diene-3,5-dione) is the main active ingredient of the yellow-orange spice, *Curcuma longa*. It is a hydrophobic polyphenol recognized as safe by the Food and Drug Administration (FDA), with a wide range of biological activities, including anti-inflammatory and antioxidant properties [13]. At the molecular level, the multiple properties of curcumin (CUR) are based on its ability to interact with many molecules due to its special chemical structure, which confers to CUR, H-bond donating/accepting capacities and multivalent cation binding properties [14]. In particular, CUR’s potent antioxidant capacity has been widely established through its ability to interact with several reactive oxygen species (ROS) (hydroxyl radical, superoxide anion, peroxyl radicals, hydrogen peroxide and singlet oxygen) [15]. In the same way, the anti-inflammatory properties of CUR have also been widely demonstrated and appear to be related to its ability to modulate several molecules in cell signaling pathways. CUR has been shown to modulate signal transducers and transcription activators like JAK-STAT, Nrf2, Notch-1 and nuclear factor kappa B (NF-kB) as well as the protein kinases Akt and MAPK [16,17]. The anti-inflammatory effects of CUR are used to target and control skin inflammation in different skin diseases; its mechanism of action involves reducing the expression of cytokines released by immune cells [18].

The efficiency of CUR in humans has been evaluated in preclinical studies and in clinical trials [19]. From 263 currently listed as completed or ongoing in “clinical-trials.gov” (accessed on 26 April 2022) only 5 are related to the topical route, where CUR was administered in conventional formulations such as gelatin capsules, solutions, gels and creams. The low number of topical studies could be related to the physicochemical characteristics of CUR such as its lipophilic character and oxygen and light instability, which limit its efficacy and clinical applications. To circumvent these limitations, CUR has been formulated in advanced delivery systems such as hydrogels [20], nanofibers [21], microsponges [22], emulsions [23], nanoparticles [24] and lipid-based nanoparticles [25], used alone or combined. In particular, a growing interest has recently been developed for nanostructured lipid carrier (NLC) formulations, which are composed of a mixture of solid and liquid lipids with a structural arrangement allowing high entrapping efficiency. Interesting curcumin–NLC delivery systems were designed for several applications. For example, optimized formulations for an intranasal administration were designed for the treatment of Alzheimer’s disease [26] showing a high drug entrapment, a sustained release profile and a good stability. For an in situ administration, Murgia et al. prepared curcumin–NLCs and placed them in the dental alveoli to prevent alveolar bone resorption following dental extractions [27]. The possibility of associating several carriers of different nature and size (nano-, micro- and bulk-scale) in a more complex system allows the accumulation of the specific properties of each carrier, the ability to obtain longer sustained release kinetics and to protect and preserve the activity of therapeutic loaded agents [28]. The combination of nanostructured lipid carriers (NLCs) and hydrogels represents a promising strategy as a dual delivery system [29]. NLCs are lipidic nanoparticles with a solid/liquid mixed core able to improve stability, entrapment efficiencies and skin penetration of hydrophobic active compounds [30]. By entrapping CUR in such nanocarriers, high entrapment efficiencies (70–85%) have been reported, increasing its apparent water solubility ~60-fold [31] and its skin penetration capability more than 3-fold [25]. In the same way, hydrogels, and especially those based in Carbopol^®^ (Lubrizol, Rouen, France), are greatly employed in the pharmaceutical and cosmetics industries. Carbopol^®^ is a polymer of acrylic acid crosslinked with allyl pentaerythritol. This gelling agent endows formulations with a transparent appearance, good suspending ability, and good sensorial and adhesion properties. Moreover, it has been reported to have low potential for skin irritation and sensitization [32] and to be biocompatible with dermal cells [33,34]. Thus, Carbopol^®^ is a good candidate for constituting the matrix of a dual system.

The current work focuses on the development and evaluation of a dual CUR-NLC/Carbopol^®^-based hydrogel delivery system for its potential use as adjuvant therapy in topical skin applications. First, entrapment of CUR into the lipid nanocarrier was studied as well as its release from it and the preservation of CUR’s antioxidant properties. Cell viability and dermal cell migration in presence of CUR-NLCs were also assessed. Next, CUR-NLCs were embedded into a Carbopol^®^-based hydrogel and its rheological properties were studied. Finally, penetration studies in Strat-M^®^ membranes were carried out with both CUR-NLCs and CUR-NLCs/Carbopol^®^-based hydrogel delivery systems.

## 2. Materials and Methods

### 2.1. Chemicals

Precirol^®^ ATO5 (gliceryl palmitostearate) and Labrafac^®^ lipophile WL 1349 (caprylic/capric triglycerides) were kindly provided by Gattefosse (Nanterre, France). Curcumin (CUR, purity ≥75% HPLC), Tween^®^ 80 (polyoxyethylene 20 sorbitan monooleate), poloxamer 407 (polyoxyethylene-polyoxypropylene copolymer), (±)-α-tocopherol (purity ≥ 75% HPLC), 2,2′-azino-bis (3-ethylbenzothiazoline-6-sulfonic acid) diammonium salt (ABTS), potassium persulfate (K_2_S_2_O_8_, purity ≥ 99%), triethanolamine (TEA, purity ≥ 99%), 3-(4,5-dimethyl-2-thiazolyl)-2,5-diphenyl-2H-tetrazolium bromide (MTT), tert-butyl hydroperoxide (Luperox^®^), 2′,7′-dichlorofluorescin diacetate (DCFH-DA) and Trolox (purity ≥ 98%) were purchased from Sigma-Aldrich (Saint-Quentin-Fallavier, France). Acetonitrile (ACN) and trifluoroacetic acid (TFA, purity ≥ 99%) were purchased from Sigma-Aldrich (Milan, Italy). Dichloromethane (DCM, purity ≥ 99.8%), ethanol (EtOH, purity ≥ 99.8%) and isopropanol (purity ≥ 98%) were purchased from VWR Chemicals (Fontenay-sous-Bois, France). Carbopol^®^ 980 NF (polyacrylic acid polymer crosslinked with allyl pentaerythritol) was kindly provided by Lubrizol (Rouen, France).

All aqueous solutions were made with ultrapure water obtained with the Milli-Q^®^ Direct Water Purification System (Merck KGaA, Darmstadt, Germany).

### 2.2. Biological Reagents

Dulbecco’s Modified Eagle Medium (DMEM), fetal bovine serum (FBS), trypan blue (TB), penicillin/streptomycin and phosphate-buffered saline (PBS) were obtained from Gibco-Fisher Scientific (Illkirch, France). Dermal Basal Medium (DBM), Keratinocyte Growth Kit (containing: bovine pituitary extract, rh TGF-α, l-glutamine, hydrocortisone hemisuccinate, rh insulin, epinephrine, and apo-transferrin), phenol red, BJ fibroblast (CRL-2522^TM^) primary cell line and the HEKn (PCS-200-010^TM^) primary cell line were purchased from ATCC^®^ (Manassas, VA, USA).

### 2.3. CUR Samples Preparation

The CUR used was a mixture of curcumin (MW 368.38 g/mol, >75% HPLC), demethoxycurcumin (MW 338.4 g/mol), and bisdemethoxycurcumin (MW 308.3 g/mol). A mean molecular weight for CUR was calculated based on the different proportions of the three individual components of CUR (see Section 3.1). The mean MW was used in all experiments for the conversion of mass concentration to molar concentration.

CUR sample solutions for physicochemical analyses were prepared in a binary solvent composed of EtOH/DCM (60/40%, *v*/*v*) [35] or in ACN when specified. Calibration curves were constructed by spectrophotometric and HPLC methods. Spectrophotometric analyses were obtained using a UVIKON-XS double beam ultraviolet–visible (UV-VIS) spectrophotometer (Secomam-Aqualabo, Champigny sur Marne, France). A UV-VIS calibration curve of CUR (1–7 µg/mL) in EtOH/DCM (60/40%, *v*/*v*) was obtained at λ = 427 nm, presenting a slope of y = (0.143 ± 0.002)x; R^2^ = 0.999. Method was tested in the range of 0.5–0.7AU. Chromatographic analyses were performed using an Azura HPLC (Knauer, Berlin, Germany) equipped with an Azura Pump P6.1L, a KnauerEurospher II C18 column (250 × 4 mm) and a UV-VIS Azura detector DAD 2.1L, controlled by ClarityChrom^®^ software version 6.1.0. The detection wavelength was fixed at λ = 425 nm. The mobile phase consisted in a 13 min-gradient from 50:50 to 0:100 (water (+0.1% TFA)/ACN (+0.1% TFA)) at a flow rate of 1 mL.min^−1^. The injection volume was 20 µL. All samples were diluted in acetonitrile (ACN) and filtrated (PTFE, 0.45 μm). An HPLC calibration curve for CUR (0.31–50 µg/mL) in ACN was performed, presenting a slope of y = (228.85 ± 3.35) x; R^2^ = 0.999. Method was tested in the range of 650–4300 mAU.s.

CUR samples for biological tests were prepared by adding 30 mg of CUR to 4.5 mL of cell culture media (DMEM 10%FBS or DBM) and stirring for 4 h at room temperature. Then samples were centrifuged at 5000 rpm for 30 min at 20 °C (Sigma 3K30 centrifuge, Sigma, Osterode am Harz, Germany. Supernatant containing dissolved CUR was separated from the pellet (undissolved CUR). Concentration of dissolved CUR was determined by dilution (1:100) in EtOH/DCM (60/40%, *v*/*v*), centrifuging the mixture for 3 min at 13,000 rpm, reading the absorbance of the supernatant at 427 nm and calculating the CUR concentration from it according to the previously established calibration curve.

### 2.4. NLCs Preparation

Unloaded NLCs (Blank-NLCs) and CUR loaded NLCs (CUR-NLCs) were prepared according to the hot homogenization method by modifying some procedures and parameters when necessary [35,36]. The general protocol was as follows. Two phases were prepared. An oil phase composed of 450 mg of Precirol^®^ ATO 5, 100 mg of Labrafac^®^ lipophile WL 1349 and 60 mg of Tween^®^ 80. For CUR-NLCs, 15 mg of CUR was added. Separately, an aqueous phase was prepared with 450 mg of poloxamer 407 dissolved in 15 mL of ultrapure water.

First, both phases were heated at 70 °C for 10 min. Secondly, the oil phase was homogenized at 10,000 rpm at 70 °C for 2 min (Polytron^®^ system, PT3100 homogenizer with a dispersing aggregate of 7 mm of diameter, Kinematica AG, Luzern, Switzerland). During this time the aqueous phase was maintained at 70 °C. Then, the aqueous phase was added to the oil phase during 1 min while the homogenization speed was increased to 20,000 rpm. Once all the aqueous phase was added and the speed reached, the homogenization was maintained for 30 min at 70 °C. Afterwards, the preparation was cooled down to 20 °C for 20 min and stored overnight at 4–8 °C. One day after preparation, the CUR loaded NLC suspension was centrifuged at 4000 rpm for 30 min at 20 °C. Pellets (corresponding to excess CUR) were separated from supernatants (CUR-NLCs). In order to remove any free molecules (lipid or surfactant) that might not have been eliminated by the centrifugation step, Blank-NLC and CUR-NLC samples were passed through a size exclusion chromatography (SEC) column. Briefly, 1 mL of the sample was deposited in a Bio-Gel^®^ P-10 polyacrylamide gel column (Bio-Rad, Marnes-la-Coquette, France). Ultrapure water was used as eluent. Dead volume was 1 mL and subsequent collected fractions were 500 µL. Fraction numbers 3, 4 and 5 were pooled for further analysis.

For Blank-NLCs, CUR was not added to the oil phase. Then, the same procedure as described above was followed.

### 2.5. NLCs Physicochemical Characterization

#### 2.5.1. Particle Size Analysis and Zeta Potential (ZP) Measurements

Particle size analysis and ZP measurements were performed using a Zetasizer Nano ZS (Malvern Instruments Ltd., Worcestershire, UK) equipped with a He-Ne laser (λ = 633 nm) at a scattering angle of 173°.

Particle size and polydispersity index (PDI) were determined by dynamic light scattering (DLS). NLC samples were diluted (1:100) in ultrapure water and placed in polystyrene cuvettes semi-micro (Brand Gmbh + Co Kg, Wertheim, Germany). Particle size was described in terms of the hydrodynamic diameter (Dh). Results were expressed as size distribution by intensity and by number. Zeta potential (ZP) was calculated from the electrophoretic mobility obtained from laser Doppler microelectrophoresis. Samples were diluted (1:10) in 1 mM KCl and measurements were performed using a folded capillary zeta cuvette (DTS1070).

In order to measure the stability of NLCs samples, DLS measurements were carried out at 1 and 7 days on NLC samples before and after the SEC column. For NLC samples before the SEC column complementary DLS, PDI and ZP measurements were carried out at 1, 7, 14, 30 and 45 days. Samples were stored at 4 °C between measurements. Each experiment was carried out at 25 °C and in triplicate (*n* = 3).

#### 2.5.2. Morphological Analysis by Transmission Electron Microscopy (TEM) Techniques

Fresh NLC samples before and after the SEC column were analyzed by transmission electron microscopy (TEM) using the negative stain method and cryo technique.

For the negative stain method, 3 µL of the NLC sample (55 mg/mL) was deposited on an air glow-discharged Quantifoil^®^ R2/2 carbon-coated grid (Quantifoil Micro Tools Gmbh, Großlöbichau, Germany) for 1 min. The excess liquid was blotted, and the grid stained with 2% *w/v* aqueous uranyl acetate. The grids were visualized at 100 kV with a Tecnai 12 Spirit transmission electron microscope (ThermoFisher, New York, NY, USA) equipped with a K2 Base 4 k × 4 k camera (Gatan, Pleasanton, CA, USA). Magnification was at 14.700 X at the level of the camera, corresponding to a pixel size at the level of the specimen of 0.34 nm.

For the cryo technique, 3 µL of the NLC sample (~60 mg/mL) was deposited on an air glow-discharged Quantifoil^®^ R2/2 carbon-coated grid (Quantifoil Micro Tools Gmbh, Großlöbichau, Germany) for 1 min. The sample excess was blotted with a filter paper, and the grid plunged into liquid-nitrogen-cooled ethane. The grid was rapidly transferred and kept under liquid nitrogen. For observation, the grids were mounted in a 626 Gatan holder using its cryo-transfer device. The observations were made in a Tecnai 200 equipped with a field-emission gun (ThermoFisher, New York, NY, USA). Images of the samples were recorded with a direct detection camera K2 Summit (Gatan, Pleasanton, CA, USA) operated in movie mode. The images were aligned and summed as recommended by the manufacturer. They were recorded at 19.800X magnification (pixel size 0.26 nm at the specimen level), using a total dose less than 20 electrons/Å2.

#### 2.5.3. Quantification of Loaded CUR in NLCs

Quantification of loaded CUR was calculated by two different approaches: the direct method and the indirect method.

For the direct method, CUR-NLC supernatants (see 2.4 NLC preparation) were dissolved and diluted (1:200) in EtOH/DCM (60/40%, *v*/*v*) and transferred to 10 mm quartz cuvettes. Complete dissolution and lack of absorption of any component of the NLCs was monitored by obtaining the absorbance spectra of Blank-NLCs and setting this as the baseline for all measurements. CUR concentration in all samples was calculated (*n* = 3) using the calibration curve of CUR in EtOH/DCM (60/40%, *v*/*v*) at λ = 427 nm as described in Section 2.3.

Encapsulation efficiency (%EE) of CUR-NLCs refers to the concentration of CUR incorporated into NLCs over the initial CUR concentration (1 mg/mL). The %EE was calculated using the following equation:

%EE = (loaded − CUR)/(total − CUR) × 100

where total–CUR is the initial CUR mass concentration introduced (1 mg/mL) and loaded–CUR represents the mass concentration of CUR loaded into the NLCs determined at the end of preparation (for a final volume of 15 mL).

Drug loading (%DL) of CUR-NLCs refers to the amount of CUR incorporated into NLCs per weight of lipids (*w*/*w*). Drug loading (%DL) was calculated using the following equation:

%DL = (loaded − CUR)/Lipids × 100

where Lipids represent the total amount of lipids used for the preparation of NLCs, including liquid (Labrafac^®^ lipophile WL 1349) and solid (Precirol^®^ ATO 5) lipids.

For the indirect method, pellets (containing excess CUR) were dissolved in ACN. Concentration of CUR in excess CUR was determined using an HPLC calibration curve of CUR as described in Section 2.3.

Encapsulation efficiency (%EE) and drug loading (%DL) of CUR-NLCs were calculated using the following equations:

%EE = ((total − CUR) − (excess − CUR))/(total − CUR) × 100

%DL = ((total − CUR) − (excess − CUR))/Lipid × 100

where excess–CUR is the quantity of CUR not loaded into the NLCs and present in the pellet. All other parameters are described in the direct method section.

#### 2.5.4. Release Studies

CUR release from CUR-NLCs was studied in PBS, DMEM (supplemented with 10% FBS) and DBM (without 10% FBS but supplemented with Keratinocyte Growth Kit, see Section 2.2 for detailed components) media in static conditions. Released CUR was indirectly determined by measurement of remaining CUR in the CUR-NLC suspension. Briefly, CUR-NLC samples (containing 20 µM of loaded CUR) prepared in the different media (4 mL) were placed in sterile conditions at 37 °C for 0, 24, 48 or 72 h incubation times. At the end of each incubation time, samples were centrifuged at 4000 rpm, 20 °C for 30 min. After centrifugation, supernatants (3 mL, containing CUR-NLCs) were freeze-dried for 24 h, then dissolved using EtOH/DCM (60/40%, *v*/*v*) and centrifuged at 9000 rpm, 4 °C for 5 min. Remaining CUR was calculated from the calibration curve after measurement of absorbance at 427 nm of these last supernatants. Released CUR was then calculated using the following equations:

Released CUR (%) = 100−Remaining CUR

Remaining CUR (%) = (CUR_t/_CUR_0_) × 100

CUR_t_: remaining CUR concentration in CUR-NLC solution at each incubation time.

CUR_0_: remaining CUR concentration in CUR-NLC solution before incubation at 37 °C (t = 0).

### 2.6. Study of NLCs Antioxidant Activity

An ABTS assay was performed on CUR, Blank-NLCs and CUR-NLCs. α-Tocopherol was used as a reference antioxidant. The procedure was adapted from previously established methodologies [35,36].

Briefly, ABTS (14 mM) and K_2_S_2_O_8_ aqueous solutions (4.9 mM) were mixed (50/50%, *v*/*v*) and placed for 24 h in the dark and at room temperature to obtain a radical cation ABTS^•+^ solution (7 mM). In order to reach an absorbance of ~0.8 at 734 nm, this ABTS^•+^ solution was diluted (1:70) in ultrapure water.

NLC samples were dissolved in EtOH/DCM (60/40%, *v*/*v*). CUR-NLCs solutions were prepared at different CUR concentrations (0–40 µM). Blank-NLC solutions were prepared at the same mass concentrations as CUR-NLCs. Solutions of CUR (0 to 277 µM) and α-tocopherol (0 to 100 µM) were prepared in the same solvent and used as controls.

Afterwards, 300 µL of these solutions was added to 1000 µL of the diluted ABTS^•+^ solution (absorbance ~0.8) and mixed for 45 min at room temperature and in the dark. Samples were centrifuged (13,000 rpm, 3 min at 4 °C) and the absorbance spectra of the aqueous phase was immediately monitored from 600 to 800 nm. Experiments were carried out in triplicate (*n* = 3).

Antioxidant activity was defined in terms of the percentage (%) of ABTS^•+^ inhibited by each sample. This was calculated using the maximal absorbance of ABTS^•+^ at 734 nm, according to the following equation:

% inhibition ABTS^•+^ = (Abs_734nm_ ABTS^•+^ − Abs_734nm_ sample)/Abs_734nm_ ABTS^•+^) × 100

α-Tocopherol equivalent antioxidant capacity (α-TEAC) was calculated from the slope ratios of the percentage of inhibition–concentration curves of respective samples and α-tocopherol (μM CUR/μM α-tocopherol).

### 2.7. Cell Culture

Human neonatal BJ fibroblasts and human epidermal keratinocytes neonatal (HEKn) primary cell lines from human foreskin were used for this study. BJ fibroblasts were cultured in DMEM supplemented with GlutaMAX already containing phenol red, complemented with FBS (10%), penicillin/streptomycin (417 U/mL) and NaHCO_3_ (0.625%). Cells were cultured in DMEM until 90–100% confluence, passages between 4 and 9 were used.

HEKn were cultured in DBM supplemented with bovine pituitary extract (0.4%), rh TGF-α (0.5 ng/mL), L-glutamine (6 mM), hydrocortisone hemisuccinate (100 ng/mL), rh insulin (5 mg/mL), epinephrine (1.0 mM), apo-transferrin (5 mg/mL), penicillin (10 U/mL) and streptomycin (10 µg/mL). Phenol red (33 µM) was added for cell culture routine procedures. For experiments where curcumin quantification was needed, phenol red was eliminated. Cells were maintained in DBM until 80% confluence, passages between 3 and 7 were used.

### 2.8. Cell Viability Evaluation

#### 2.8.1. MTT and TB Assays

The MTT assay and the trypan blue (TB) exclusion assay were used to study the in vitro effect of CUR, CUR-NLCs and Blank-NLCs on BJ fibroblasts and HEKn cell lines.

For the MTT test 50,000 cells/well were seeded in a Corning^®^ 96-well plate with a clear bottom (New York, NY, USA) and incubated for 24 h (37 °C and 5% CO_2_). Then, the medium was removed, cells were washed (PBS) and treated (24 h) with 10% FBS DMEM or DBM with or without samples: CUR (1–20 µM), CUR-NLCs (1–20 µM of loaded CUR corresponding to 0.05–1.10 g/L of NLCs suspension) or Blank-NLCs (0.05–1.10 g/L). After washing, MTT (200 µL, 0.5 g/L in PBS) was added and incubated for 2 h 30 min at 37 °C and 5% CO_2_. MTT was then removed and isopropanol (200 µL) was added to each well for 40 min. Absorbance at 570 nm was measured using a microplate reader (Xenius XM, Safas, Monaco). Metabolic activity (%) of cells treated with the samples (experimental cells) was reported compared with the metabolic activity of those treated with only 10% FBS DMEM or DBM (control cells, 100% metabolic activity). Experiments were performed in triplicate. The following equation was used:

% metabolic activity = (Abs_570nm_ experimental cells/Abs_570nm_ control cells) × 100

For the TB exclusion assay, cells were seeded in a Corning^®^ 24-well plate with a clear bottom (New York, NY, USA) and cultured 24 h. Then, cells were incubated without samples (control) or with samples (24 h) at the same concentration as described for the MTT test. Cells were detached, stained with TB (25 µL at 0.04% *v*/*v* in PBS) and live/dead cells counted in a Malassez chamber using a DMi1 inverted microscope (Leica Microsystems, Wetzlar, Germany). Blue cells were counted as dead. Control cells represented 100% viability. Experiments were performed in triplicate.

#### 2.8.2. Evaluation of BJ Fibroblasts under Oxidative Stress

For oxidative cell stress induction evaluation different Luperox^®^ (stressor) concentrations were used to induce ROS production. ROS generation was demonstrated using the fluorogenic probe DCFH-DA. For this, 50,000 cells/well (156,000 cells/cm^2^) were seeded in a 96-well plate in 10% FBS DMEM medium for 48 h. Medium was removed and cells were incubated with 100 µL of DCFH-DA 20 µM for 1 h. Next, cells were washed with PBS and 100 µL of Luperox^®^ at 50, 100 and 200 µM was added. Fluorescence (at λexcitation = 485 nm/λemission = 530 nm) was recorded during 180 min using a Synergy HTX Multi-Mode Reader (BioTek^®^ Instruments, Vermont, USA) set at 37 °C. Samples without probe, either with or without stressor were used as blanks.

For cell viability under stress conditions: cells were seeded in a 96-well plate at a concentration of 50,000 cells/well (156,000 cells/cm^2^) in 10% FBS DMEM medium. Plates were incubated for 24 h at 37 °C and 5% CO_2_. Medium was taken off and cells were washed with 200 µL of PBS. Then, cells were treated with Luperox^®^ at 50, 100, 200 or 300 μM for 1 h. After induction of stress, Luperox^®^ was removed and the MTT test was performed as follows: 200 µL of MTT 0.5 g/L in PBS was added to experimental wells, then incubated for 2 h 30 min at 37 °C and 5% CO_2_. MTT was then removed and isopropanol (200 µL) was added to each well for 40 min. Absorbance at 570 nm was measured using a microplate reader (Xenius XM, Safas, Monaco). The % Metabolic activity was calculated as described in Section 2.8.1. Experiments were performed in triplicate. Photos were taken after incubation with MTT using a DMi1 inverted microscope (Leica Microsystems, Wetzlar, Germany). Obtained RGB images were converted to grayscale images using Fiji software (ImageJ, NIH, USA, version 2.0.0.).

#### 2.8.3. Effect of NLCs on BJ Fibroblasts under Stress Conditions

The influence of NLCs on cell viability was also studied under stress conditions for BJ fibroblasts. They were seeded in a 96-well plate at a concentration of 50,000 cells/well (156,000 cells/cm^2^) in 10% FBS DMEM medium. Plates were incubated for 24 h at 37 °C and 5% CO_2_. Medium was taken off and cells were washed with 200 µL of PBS. After 24 h incubation with 10 µM CUR-NLCs, 0.54 g/L Blank-NLCs or Trolox at 10 µM in 10% FBS DMEM, medium was removed and cells were washed with 200 µL of PBS. Then, cells were treated with Luperox^®^ at 100 μM for 1 h. Afterwards, Luperox^®^ was removed and the MTT test was performed as in no stress conditions: 200 µL of 0.5 g/L MTT in PBS was added to experimental wells and then plates were incubated for 2 h 30 min (37 °C and 5% CO_2_). After incubation, photos were taken using a DMi1 inverted microscope (Leica Microsystems, Wetzlar, Germany). Obtained RGB images were converted to grayscale images using Fiji software (ImageJ, NIH, USA). MTT was then removed and 200 µL of isopropanol was added to each well. Plates were gently stirred and stored at room temperature in the dark for 40 min. The percentage of viable cells was calculated as in no stress conditions through absorbance readings at 570 nm as described in Section 2.8.1 with a microplate reader (Xenius XM, Safas, Monaco).

### 2.9. Cell Migration/Proliferation Assay

Silicone culture inserts (Ibidi, Martinsried, Germany) were used to evaluate the migration/proliferation of dermal cell lines. The protocol was carried out as reported in the literature, with some modifications when needed [37]. Briefly, cells were seeded at a density of 159,000 cells/cm^2^ (35,000 cells per well) for BJ fibroblasts and 95,500 cells/cm^2^ (21,000 cells per well) for HEKn in the 2-well culture inserts that were previously attached to the wells of a 12-well plate. After 24 h, inserts were taken off, leaving a cell-free gap. Cells were washed twice with 2 mL of PBS. Then, 2 mL of 10% FBS DMEM or DBM containing CUR (0–20 µM) or CUR-NLCs (1–20 µM of loaded CUR in 0.05–1.10 g/L of NLC suspension) or Blank-NLCs (0.05–1.10 g/L) was added to the cells. Phase-contrast images of the gap were taken immediately after adding the treatment using a DMi1 inverted microscope (Leica Microsystems, Wetzlar, Germany). Cells were incubated for 24 h and photos were taken again in the same place. For each photo, the areas were measured using the manual area measurement tool in the Fiji software (ImageJ, NIH, USA). For each sample, 5 images corresponding to different places along the gap were analyzed. Experiments were repeated twice. Pixel size at the specimen level was 0.235 µm.

For controls, cell medium (10% FBS DMEM or DBM, for BJ fibroblasts and HEKn, respectively), was used without CUR, Blank-NLCs or CUR-NLCs.

Reduction in the gap area was determined by comparing the areas in the photos at 0 h with the ones at 24 h with the following equation:

% Reduction of gap area = 100 − [(Area at 24 h_/_Area at 0 h) × 100]

### 2.10. Preparation of NLCs Loaded Gel

Carbopol^®^ 980 NF was dispersed at 0.69%, *w/v* in ultrapure water by overnight stirring at room temperature. A total of 25 µL of TEA 20%, *v/v* was added for each mL of the final volume of the gel to be prepared and mixed until obtaining a homogenous mixture. The pH was ensured to be around 7. Then, Blank-NLCs (to form Blank-NLC gels) or CUR-NLCs (to form CUR-NLC gels) were added at 25% *v/v* of the final target volume of gel. The mixture was stirred and the final pH was checked to be around 6.4. To prepare the unloaded gel, NLCs were replaced by ultrapure water. The final Carbopol^®^ 980 NF concentration in the gel was 0.50% *w*/*v*.

### 2.11. Rheological Characterization of NLCs Loaded Gel

All characterization was performed using a stress-controlled rheometer (Discovery HR-1, TA Instruments, New Castle, DE, USA) joined to a cone-plane geometry (diameter: 40 mm, truncation gap: 27 µm and cone angle: 1.005°). Results were obtained with Trios V.4.7 software (TA Instruments, New Castle, DE, USA). Every analysis was performed at 32 °C (the temperature on the surface of the skin). For each condition, experiments were repeated three times (*n* = 3).

In order to determine the linear viscoelastic region (LVE), strain sweep experiments were carried out at 1 Hz. To study the behavior of the formulations when applied and rubbed on the surface of the skin, flow curves of the viscosity in function of the shear rate (from 0.001 s^−1^ to 1000 s^−1^) were constructed.

A strain recovery test was performed in order to mimic the behavior of the formulations during application. For this, viscosity of the sample was continuously monitored while applying intercalated low and high shear cycles. Starting with a low shear cycle, a total of five cycles were applied during each single test. The three low shear cycles were performed at 0.1 s^−1^ for 150 s and the two high shear cycles at 100 s^−1^ for 30 s.

### 2.12. Penetration Studies with CUR-NLCs and CUR-NLCs/Gel

Strat-M^®^ membranes (25 mm) were obtained from Merck KGaA (Darmstadt, Germany). Due to their multi-layer composition, they have proven to be good at mimicking intact skin barrier properties [38,39]. Immediately after unpacking, each membrane was clamped between the donor and the receptor compartment of a vertical Franz diffusion cell of 9 mm diameter. The donor and receptor compartments were filled with 5 and 1 mL PBS at pH 5.5 (pH regulated with HCl 1 M), respectively, and placed in a water bath at 32 °C, to mimic the temperature at the surface of skin, and under stirring at 290 rpm. After 10 min, PBS was removed from the donor compartment and replaced with either 1 mL of CUR-NLC suspension in ultrapure water containing 122 ± 4 μg of CUR, or 1 g of CUR-NLCs/gel containing 125 ± 4 μg of CUR, prepared as described in Section 2.10. After 15 min, 30 min, 1 h and 24 h of incubation, membranes were removed, excess CUR-NLCs and gel materials were removed with a clean paper and membranes were washed 3 times with ultrapure water. Excess water was removed by blotting, membranes were cut into small pieces and kept overnight under magnetic stirring in determined volumes of ACN/H_2_O (50/50%, *v*/*v*) in order to extract penetrated CUR: 2 mL for CUR-NLCs/gel samples and CUR-NLC samples after 15 min; 3 mL for CUR-NLC samples after 30 min; 5 mL for CUR-NLC samples after 1 h and 9 mL for CUR-NLC samples after 24 h. Samples were then centrifuged at 4000 rpm for 10 min and supernatant was analyzed using HPLC for determining the amount of CUR penetrated into the membranes (Q_T_). Throughout the diffusion experiments 1 mL was removed from the receptor compartment every 1 h and analyzed by DLS as specified in Section 2.5.1. Volume taken off was replaced by an equal volume of PBS at pH 5.5.

The amount of CUR remaining in the donor compartment was determined at the end of each timepoint. For that, CUR-NLC suspension (0.5 mL) or CUR-NLCs/gel (500 mg) were collected from the donor compartment. Samples were freeze-dried, dissolved in 4 mL of EtOH/DCM (60/40%, *v*/*v*) and then centrifuged at 4000 rpm for 15 min. Absorbance was measured by UV-VIS spectrophotometry. All experiments were performed in triplicate (*n* = 3).

From the amount of CUR penetrated into membranes (Q_T_), the amount of drug entrapped per unit area (De) was calculated as follows [40]:

De = Q_T_/A

where A is the area available for penetration (2.54 cm^2^)

The accumulation (Ac) was then calculated by:

Ac = De/Cd

where Cd is the starting CUR mass concentration loaded in the donor compartment

### 2.13. Statistical Analysis

The software OriginPro 2018 (Northampton, MA, USA, version 9.5) was used. All results are presented as mean ± standard deviation (SD). For the MTT test, the analysis of statistical significance was performed using one-way analysis of variance (ANOVA) followed by Tukey’s multiple comparison HSD (honestly significant difference) post hoc test. For penetration studies, analysis of the statistical significance was performed using two sample Student’s *t*-test.

## 3. Results

### 3.1. Preparation and Quantification of CUR-NLCs

**Figure 1:**
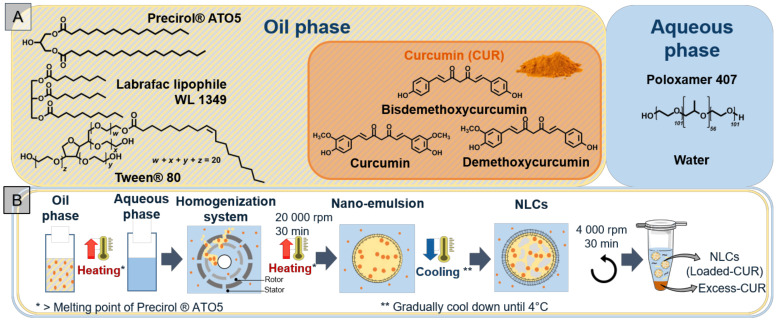
(**A**) Components of NLCs: in yellow and grey stripped box, lipids (solid and liquid), co-surfactant and CUR, composed of three curcuminoids (orange box); in blue box, aqueous phase containing the surfactant. (**B**) NLCs preparation by hot homogenization method at heating temperature of 70 °C and separation of non-loaded CUR (Excess-CUR) by centrifugation. (Created with BioRender.com (accessed on 26 April 2022)).

The oil phase, composed of the solid (Precirol^®^ ATO 5) and the liquid (Labrafac^®^ lipophile WL 1349) lipids into which the CUR and the co-surfactant (Tween^®^ 80) are introduced, is mixed with the aqueous solution that contains the surfactant (poloxamer) (Figure 1A).

**Figure 2:**
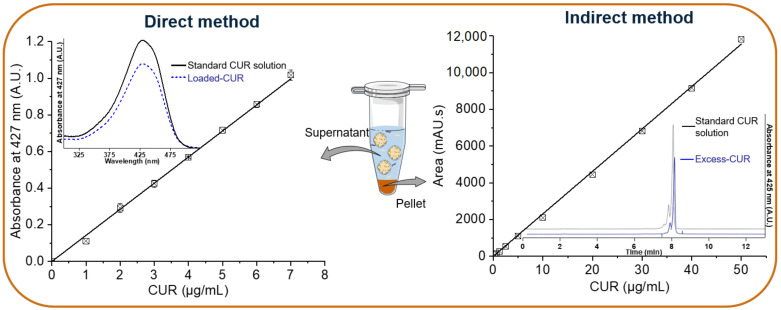
Quantification of loaded CUR in NLCs by the direct and indirect methods. For direct method NLCs were dissolved in a binary solvent composed of EtOH/DCM (60/40% *v*/*v*) before spectrophotometric analysis. For indirect method excess CUR was dissolved in ACN and determined by HPLC-DAD. (Created with BioRender.com (accessed on 26 April 2022)).

NLC aqueous suspensions were obtained by the hot homogenization method (see Section 2.4). For CUR-NLCs, total CUR introduced into the preparation is distributed between the NLCs (Loaded-CUR) and the pellets (Excess-CUR) (Figure 1B). NLCs remain in the supernatant after centrifugation. For the direct quantification method, the supernatant is diluted with a binary solvent composed of EtOH/DCM (60/40%, *v*/*v*). This binary solvent was chosen for its high solubility power for lipids [35] and used for all spectrophotometric measurements. The standard CUR and loaded CUR show the same absorption spectra with a λmax = 427 nm. The CUR calibration curve gives an extinction coefficient of ε427 nm = 0.143 μg^−1^.mL (Figure 2, direct method). For the indirect quantification method, the pellet was dissolved in ACN for HPLC analysis. The standard CUR and Excess-CUR chromatograms present the same peaks corresponding to the three components of CUR: bisdemethoxycurcumin (retention time, Rt of 7.55 min), demethoxycurcumin (Rt 7.79 min) and curcumin (Rt 8.04 min). The percentages of these three components of CUR were determined from relative area peaks: curcumin (79%), demethoxycurcumin (19%) and bisdemethoxycurcumin (2%). These results confirm the purity given by the supplier and allow us to calculate a mean molecular weight for CUR: MW 361.4 g/mol. Chromatograms and CUR calibration curves based on the three peaks integration is presented in Figure 2, indirect method.

Table 1 shows results of CUR quantification in NLCs obtained before the SEC column step. Similar results for loaded CUR were obtained with the direct and indirect methods (0.85 mg/mL and 0.83 mg/mL, respectively), showing the accuracy of the UV-VIS spectrophotometry method. After preparation of CUR-NLCs, nearly 85% of CUR was encapsulated (%EE, Table 1). A CUR drug loading (%DL) of approximately 2.3% indicates the predominance of lipid nature in CUR-NLCs. This highlights the importance of taking unloaded Blank-NLCs as a control group for antioxidant and biological experiments in order to get an accurate understanding of the effects of the loaded drug. Results of CUR quantification in NLCs obtained by the direct method (UV-VIS) before and after SEC column purification are compared in Table 2. When comparing CUR quantification (loaded CUR and EE%) in NLCs before and after the SEC column, a ratio of ~1.5–2 is found (0.85 mg/mL vs. 0.44 mg/mL, for loaded CUR and 84.56% vs. 54.79%, for EE%). These results could be explained in part by the fact that only fraction numbers 3, 4 and 5 were pooled (see Section 2.4).

### 3.2. Physicochemical Characterization

**Figure 3:**
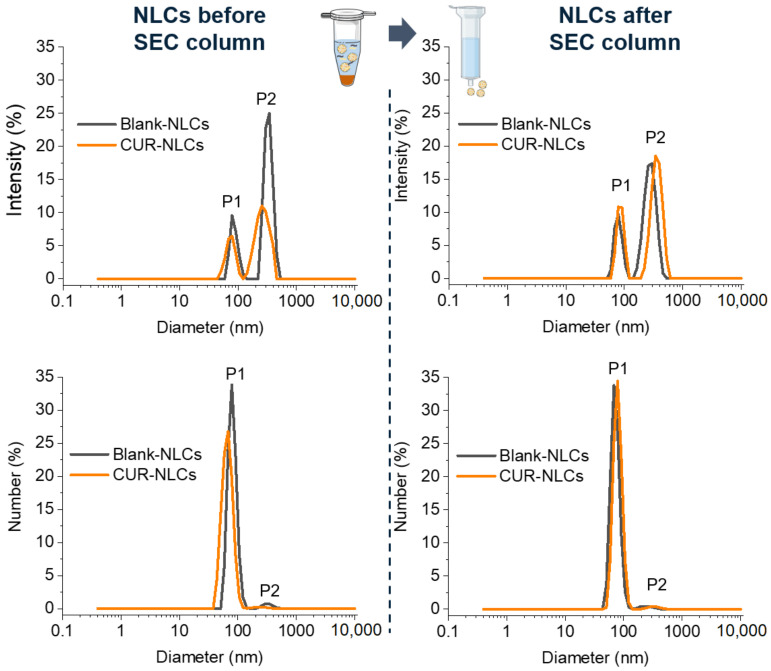
Particle size analysis in % intensity and in % number of Blank-NLC and CUR-NLC suspensions before and after passing through the SEC column. (Created with BioRender.com (accessed on 26 April 2022)).

NLCs samples were characterized after 1 day and before and after the SEC column (Table 3, values of day 1). Particle size analysis, expressed as % of intensity or number, evidenced two populations of NLCs: a small-size-NLC population (P1, 70–90 nm) and a large-size-NLC population (P2, 300–350 nm). Distribution of these two populations was ~20% (P1) and ~80% (P2) expressed as % of intensity. However, when NLC size was expressed as % of number, the small-size-NLC population, P1, represented the principal peak (Figure 3). In all cases PDI was between 0.34–0.44. Blank-NLCs and CUR-NLCs present similar size and distribution profiles. ZP measurements showed a slight difference in the NLC surface charge before and after the SEC column (~−9 mV and ~−17 mV, respectively).

**Figure 4:**
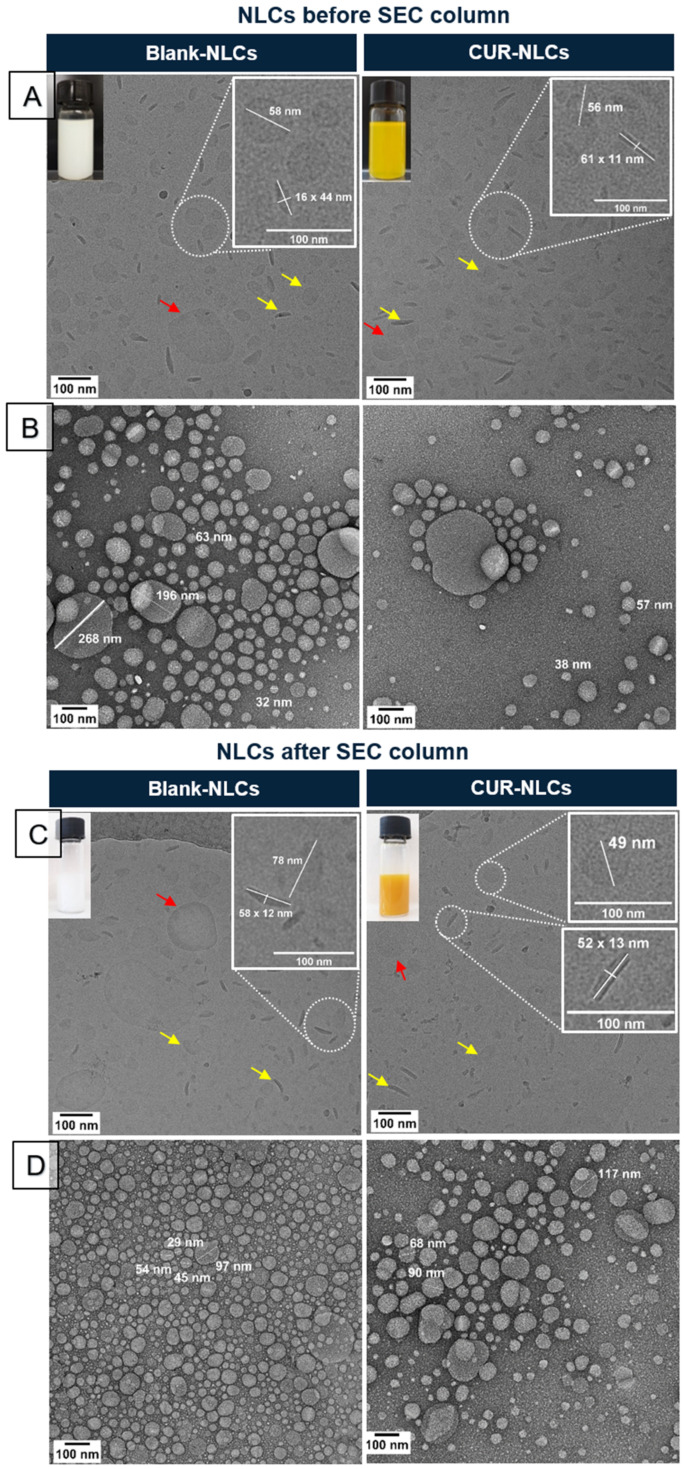
Morphological characterization of Blank-NLC and CUR-NLC suspensions. (**A**) Visual aspect and cryo-TEM images of samples before passing through the SEC column. (**B**) TEM images of samples before passing through the SEC column and after negative staining with uranyl acetate 2% *v/v* in water. (**C**) Visual aspect and cryo-TEM images of samples after passing through the SEC column. (**D**) TEM images of samples after passing through the SEC column and after negative staining with uranyl acetate 2% *v/v* in water. Yellow arrows show the ovoid platelet (top view) or rod (side view) shapes of small-size-NLC population. Red arrow shows the top view of large-size-NLC population.

Ovoid shape NLCs were observed by cryo-TEM and negative-stain-TEM analyses (Figure 4A,C and Figure 4B,D, respectively). Observations confirmed the polydispersity and the presence of two size populations in Blank-NLCs and CUR-NLCs. Analyses by the cryo-TEM technique showed that small-size NLCs adopt two preferential conformations: an ovoid shape conformation when observed by the top view and a rod shape when NLCs are observed by the side view (yellow arrows in Figure 4A,C). Only ovoid shape conformation was observed for large-size NLCs (red arrows in Figure 4A,C). These results point to a main ovoid platelet shape in NLCs. Some particles are surrounded by a darker edge, in both the top and the side view (red and yellow arrows on Blank-NLCs after the SEC column, in Figure 4C). A small compartment seems to be present inside or at the surface of some particles (Figure 4B,D).

**Figure 5:**
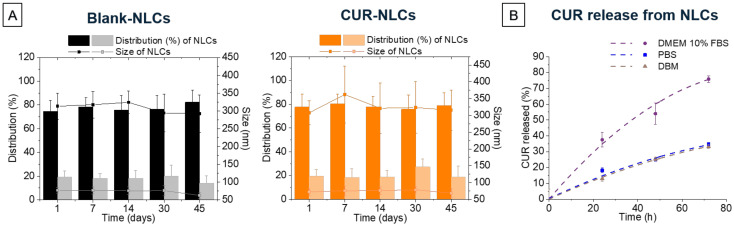
NLCs stability and CUR release. (**A**) Stability of Blank-NLCs and CUR-NLCs before SEC column at 1, 7, 14, 30 and 45 days. Bar graphs indicate the distribution in % intensity of NLCs, while line and dot graphs indicate the size of each NLC population in terms of their diameter. (**B**) CUR release from NLCs (after SEC column) in different biological media.

Stability studies of NLC samples were carried out after 7 days on both NLC samples before and after the SEC column (Table 3, values of day 7). DLS measurements show that size did not significantly change over this period of time for Blank-NLCs or CUR-NLCs (Table 3). Considering the possibility of storing NLC samples until their SEC column purification, further studies about the stability of NLC samples before the SEC column were carried out. DLS measurements were carried out at 1, 7,14, 30 and 45 days after preparation of NLC samples before the SEC column. Results show that size and distribution do not significantly change over time (Figure 5A).

Figure 5B represents CUR release from NLCs (after SEC column) in different biological media without proteins (PBS) and with proteins (10% FBS DMEM and supplemented DBM). Delivery kinetics are the same for PBS and DBM, with a 35% CUR release after 72 h. In 10% FBS DMEM, release is slightly more than two times higher (75% after 72 h).

### 3.3. Study of NLCs Antioxidant Activity

**Figure 6:**
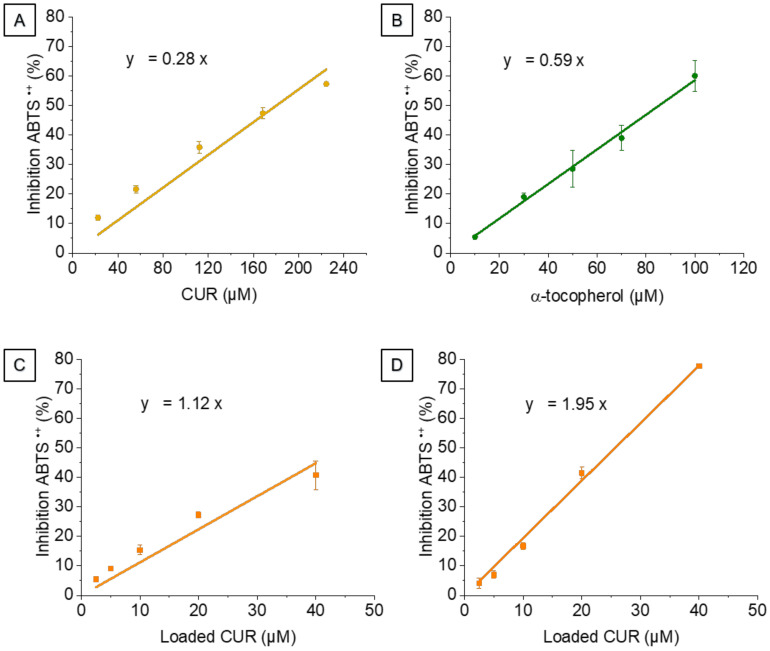
Inhibition of ABTS^•+^ at different antioxidant concentrations. (**A**) CUR. (**B**) α-Tocopherol, antioxidant standard. (**C**) Loaded CUR in CUR-NLCs before SEC column. (**D**) Loaded CUR in CUR-NLCs after SEC column.

Inhibition curves for CUR (Figure 6A) and CUR-NLCs before (Figure 6C) and after the SEC column (Figure 6D) were obtained by plotting the ABTS^•+^ inhibition % vs. the concentration of CUR. α-Tocopherol (Figure 6B) was used as an antioxidant standard. A background activity was observed for Blank-NLCs, which was subtracted from the activity of CUR-NLCs in order to only take into account the antioxidant activity of CUR.

The inhibition % of ABTS^•+^ was proportional to the CUR concentration in NLCs up to 50 μM (Figure 6C). For higher CUR concentrations in NLCs before the SEC column a saturation activity was observed with an inhibition plateau (data not shown). This saturation effect was not observed at the same range concentrations for the other formulations (CUR, CUR-NLCs after the SEC column and α-tocopherol).

Figure 6A shows the antioxidant activity of CUR and has a slope of 0.28. Higher slopes of CUR-NLCs before and after the SEC column were found when compared with CUR (1.12 and 1.95, respectively, Figure 6C,D). Table 4 shows the α-TEAC scores, calculated as α-tocopherol equivalents (µM CUR/µM α-tocopherol).

### 3.4. NLCs Biological Characterization

#### 3.4.1. Cell Viability Evaluation—MTT and TB Assays

**Figure 7:**
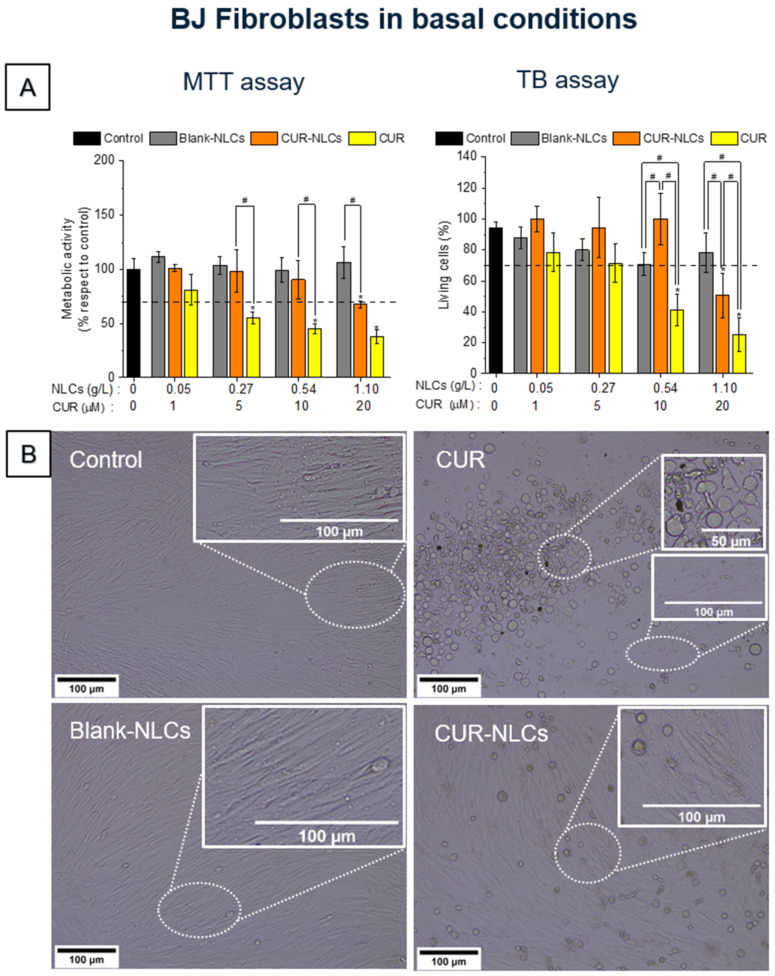
Cell viability assays on the BJ fibroblast cell line in basal conditions after 24 h treatment with CUR, CUR-NLCs or Blank-NLCs. (**A**) MTT and TB assays on BJ fibroblasts. (**B**) Phase contrast images of BJ fibroblasts after 24 h treatment with 10% FBS DMEM alone as control; CUR at 20 µM; Blank-NLCs at 1.1 g/L; or CUR-NLCs at 1.1 g/L containing 20 µM of loaded CUR. Asterisks denote statistically significant differences between an experimental group and the control group, while hashes denote statistically significant differences between two experimental groups. One-way analysis of variance (ANOVA) followed by Tukey’s multiple comparison HSD post hoc test were carried out and statistically significant differences were identified when *p*-values were lower than 0.05 (* *p* < 0.05 or # *p* < 0.05).

**Figure 8:**
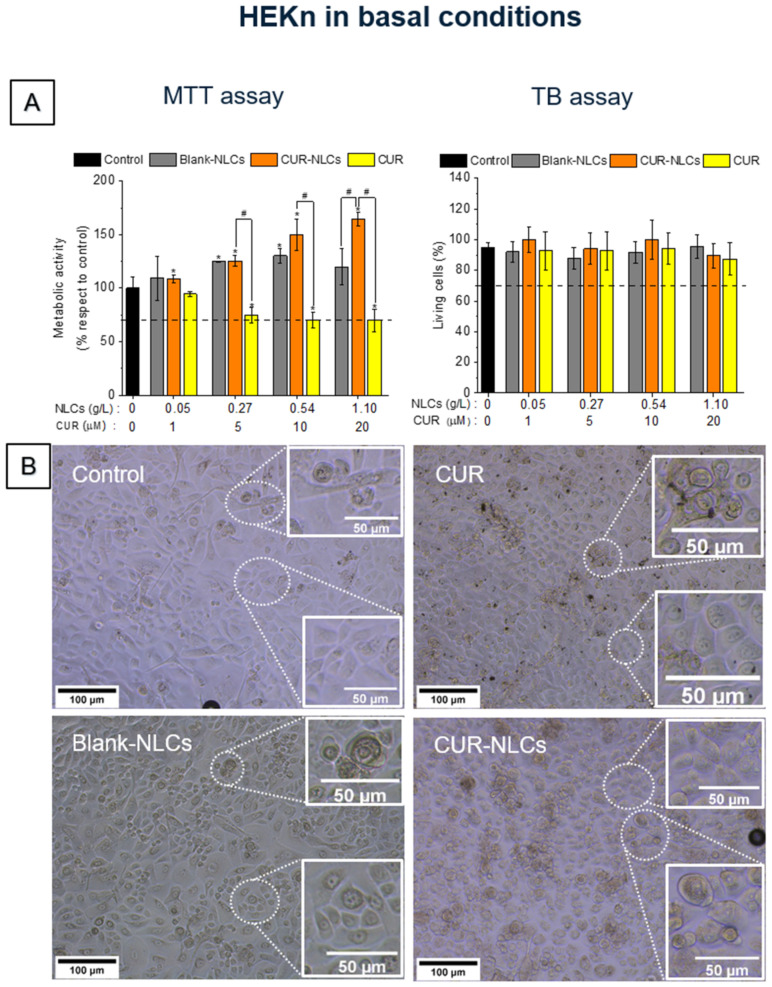
Cell viability assays on the HEKn cell line in basal conditions after 24 h treatment with CUR, CUR-NLCs or Blank-NLCs. (**A**) MTT and TB assays on HEKn. (**B**) Phase contrast images of HEKn after 24 h treatment with 10% FBS DMEM alone as control; CUR at 20 µM; Blank-NLCs at 1.1 g/L; or CUR-NLCs at 1.1 g/L containing 20 µM of Loaded CUR. Asterisks denote statistically significant differences between an experimental group and the control group, while hashes denote statistically significant differences between two experimental groups. One-way analysis of variance (ANOVA) followed by Tukey’s multiple comparison HSD post hoc test were carried out and statistically significant differences were identified when *p*-values were lower than 0.05 (* *p* < 0.05 or # *p* < 0.05).

The evaluation of the effects of CUR, CUR-NLCs and Blank-NLCs on cellular metabolism and viability was performed on BJ fibroblasts (Figure 7) and HEKn cells (Figure 8) using the MTT and TB methods. In the MTT test, a tetrazolium salt is reduced mainly by mitochondrial dehydrogenases in metabolically active cells, giving rise to a purple formazan product whose concentration can be quantified through absorbance measurements [41]. The TB test is based on the principle that the membranes of living cells do not allow the passage of certain compounds, including dyes such as trypan blue. Incubated with the dye, living cells therefore remain colourless, while the weakening of the membrane in dead cells allows the passage of the dye and the cells therefore become blue.

In the case of BJ fibroblasts (Figure 7), after 24 h treatment with CUR (≥ 5 µM of CUR), cells showed a loss of metabolic activity and an important diminution in the number of living cells compared with the control group (*p* < 0.05). Treatment of BJ fibroblasts with CUR-NLCs or Blank-NLCs did not induce cellular behavior changes compared with the control group. However, cells cultured with 20 µM CUR-NLCs showed a diminution in their metabolic activity and viability (*p* < 0.05).

Treating HEKn cells with CUR (≥5 µM of CUR) induced a loss of metabolic activity, which was visible after 24 h (Figure 8, MTT assay) (*p* < 0.05). However, the TB test showed a number of living cells (Figure 8, TB assay) comparable to the control cells. Surprisingly, the treatment of HEKn cells with CUR-NLCs or Blank-NLCs induced an increase in cellular metabolism compared with the control group. The cellular viability was found comparable to the control.

Taking into account the ISO 10993–1:2018 norm criteria regarding the evaluation of medical devices, Blank-NLCs have no toxic effect up to 1 g/L (20 µM of CUR) on both studied dermal cell lines. CUR-NLCs present no cytotoxicity at concentrations ≤20 µM of CUR on HEKn cells and ≤10 µM of CUR on BJ fibroblasts. In contrast, the tested CUR concentrations (≥5 µM) might be classified as cytotoxic as the cell viability is ≤70% compared with the control.

#### 3.4.2. BJ Fibroblasts under Stress Viability Evaluation

**Figure 9:**
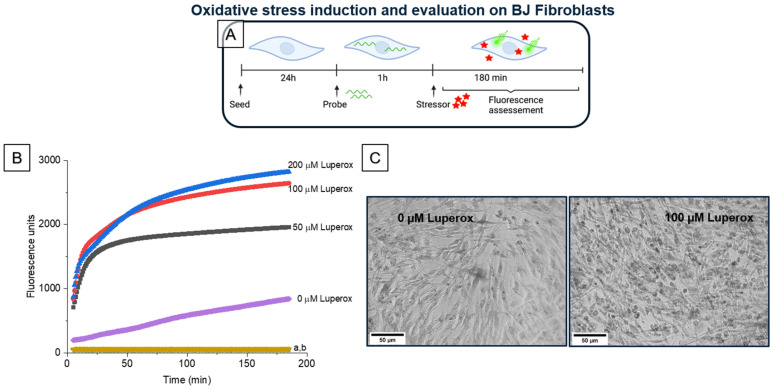
Evaluation BJ fibroblasts under oxidative stress. (**A**) In vitro stress model for the induction and the evaluation of oxidative stress. (**B**) Evaluation of oxidative stress induced by different concentrations of the stressor (Luperox). a: Cells exposed to the probe but not the stressor. b: Cells exposed to neither the probe nor the stressor. (**C**) Phase contrast images of BJ fibroblasts after 1 h treatment with 0 µM or 100 µM of stressor and subsequently incubated with MTT for 2 h 30 min. (Created with BioRender.com (accessed on 26 April 2022)).

**Figure 10:**
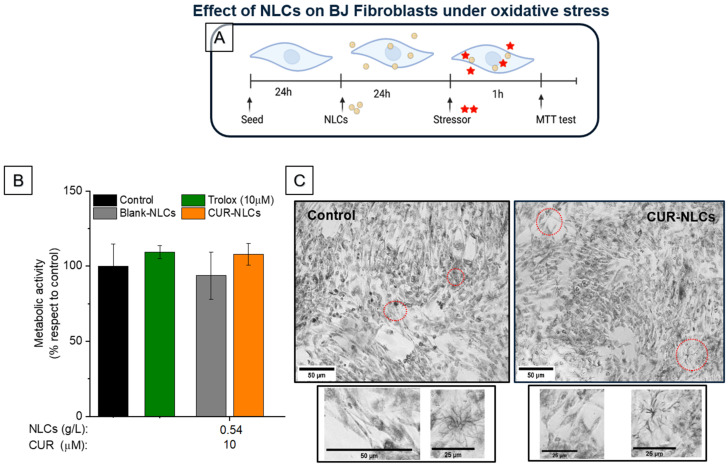
Effects of NLCs on BJ fibroblasts under oxidative stress. (**A**) In vitro model for the evaluation of the effect of NLCs on BJ fibroblast metabolic activity after undergoing oxidative stress for 1 h. (**B**) Impact of Blank-NLCs and CUR-NLCs on the metabolic activity of BJ fibroblasts undergoing oxidative stress; Trolox 10 µM was used as an antioxidant standard. (**C**) Phase contrast images of BJ fibroblasts after treatment with 10% FBS DMEM alone as control, or 0.54 g/L of CUR-NLCs containing 10 µM of CUR for 24 h prior to exposure to 100 µM of stressor for 1 h and subsequent incubation with MTT for 2 h 30 min. Images in the lower panel correspond to magnifications of the indicated zones in the upper part of the images (red circles). Extended morphology of fibroblasts as well as the formation of formazan crystals can be distinguished, evidencing metabolically active cells. (Created with BioRender.com (accessed on 26 April 2022)).

Oxidative stress can be induced in cells and tissues by different types of stressors that trigger different levels of toxicity in cells according to their chemical composition but also according to their concentration. In this work, we chose to evaluate the induction of oxidative stress by tert-butylhydroperoxide (Luperox^®^). In order to find the optimal concentrations of Luperox^®^ that do not affect the morphology or metabolic activity of the cells, increasing concentrations of the stressor were added to the cells. Then, the intracellular kinetics of ROS production was evaluated by measuring the fluorescence responsiveness of the probe, DCFH-DA, for 180 min (Figure 9A). Results show that the concentration of 100 μM Luperox^®^ induced a significant fluorescent signal (Figure 9B), corresponding to the ROS production by cells. In parallel, the viability of BJ fibroblasts under the same Luperox^®^ concentration was confirmed by an MTT assay (Figure 9C and Figure 10B (control)).

With the aim of evaluating BJ fibroblasts metabolism under stress conditions in response to the presence of NLCs loaded or not with CUR, we assessed the fluorescence reactivity of the probe to Luperox^®^ at 100 μM. The cells were pretreated with Blank-NLCs or CUR-NLCs (24 h) before the stress induction; for the positive control we treated the cells with a reference antioxidant, Trolox; for the negative control the cells did not receive any antioxidant treatment (Figure 10A). Results show that the exposure of cells to NLCs (0.54 g/L or 10 µM of CUR) does not induce significant changes in metabolism or cell morphology (Figure 10B,C).

#### 3.4.3. Cell Migration/Proliferation Studies

**Figure 11:**
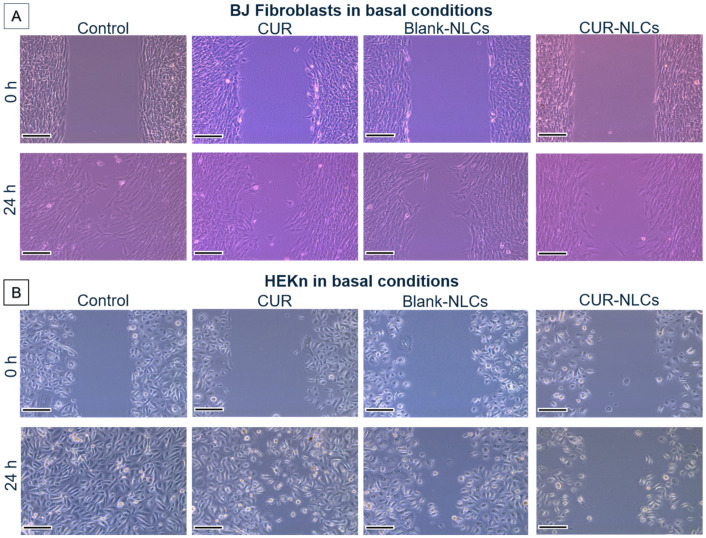
Cell migration/proliferation studies on BJ fibroblasts and on HEKn cell lines in basal conditions and under treatment with CUR (5 µM), Blank-NLCs (0.27 g/L) or CUR-NLCs (0.27 g/L containing 5 µM of loaded CUR). (**A**) Phase contrast images of the gap at 0 h and after 24 h of treatment for BJ fibroblasts. Control group was treated with 10% FBS DMEM. (**B**) Phase contrast images of the gap at 0 h and after 24 h of treatment for HEKn cells. Control group was treated with DBM. Scale bar: 100 µm.

The capacity of BJ fibroblasts (Figure 11A) and HEKn cells (Figure 11B) to migrate and or proliferate after 24 h of culture were evaluated using an in vitro model [37]. Cell motility and proliferation were evaluated by image analysis according to the capacity of cells to migrate and fill the cell-empty gap initially created. Results were expressed as gap reduction (%) after 24 h.

Effects of CUR (5 µM), CUR-NLCs (5 µM) and Blank-NLCs (0.27 mg/mL, corresponding to the same lipid content of that in CUR-NLCs at 5µM) were studied and compared with the controls (cell medium without CUR or NLCs) (Figure 11).

Figure 11A shows that after 24 h of culture (control conditions), BJ fibroblasts were able to migrate and reduce the empty zones in the culture plate (53 ± 7% of gap reduction). Similar results were found after 24 h of cell treatment with CUR or Blank-NLCs (52 ± 5% and 50 ± 9% gap reduction, respectively), whereas with CUR-NLCs, a moderate reduction in cell motility was observed compared with the control (35 ± 3% of gap reduction). None of the treatments (CUR, Blank-NLCs or CUR-NLCs) seemed to affect the morphology of the BJ fibroblasts compared with the control.

For migration studies on HEKn cells (Figure 11B), an important cell migration/proliferation capacity with a complete reduction of the gap (100 ± 3% of gap reduction) under cell control conditions was observed. A similar result was obtained for cells treated for 24 h with CUR or Blank-NLCs (90 ± 8% and 96 ± 6% of gap reduction, respectively). When CUR-NLCs were added to the medium and put in contact with the HEKn cells, a moderate decrease in cell migration capacity was observed (80 ± 4% of gap reduction). This represents a diminution of 20% in cell migration in the presence of CUR-NLCs for both the BJ fibroblast and HEKn cell lines.

However, in the case of HEKn cells, cell morphology is altered by the presence of CUR, Blank-NLCs and CUR-NLCs, displaying a rounder shape compared with the control in all cases. It should be noted that at these NLC concentrations (0.27 mg/mL, corresponding to 5 µM of CUR) neither Blank-NLCs nor CUR-NLCs exhibit cell toxicity, as shown in Figure 8A. In the MTT assay, an increase in the metabolic activity due to NLCs is even is observed.

### 3.5. Preparation and Rheological Characterization of NLCs Loaded Gel

**Figure 12:**
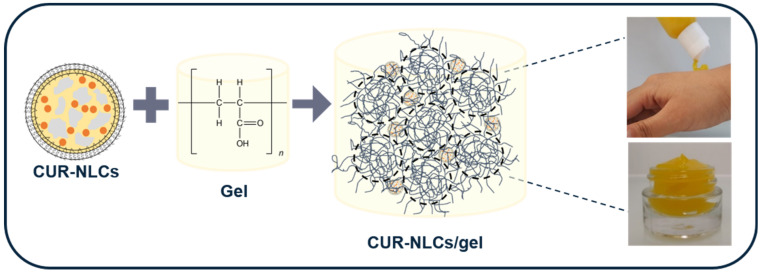
Formulation of the multiscale platform NLCs in gel.

CUR-NLCs after SEC column purification were incorporated in a Carbopol^®^ 980 gel matrix in order to form a dual platform for CUR delivery to the skin (Figure 12).

**Figure 13:**
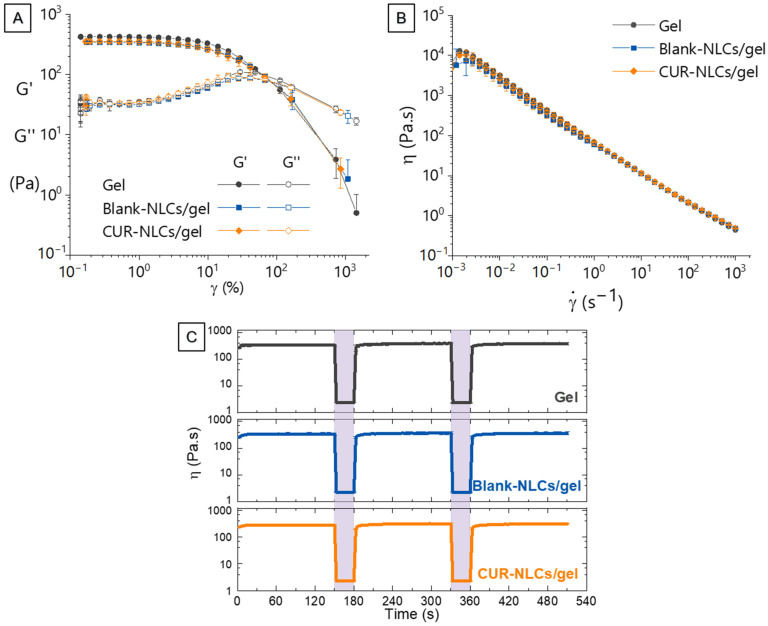
Rheological characterization of the multiscale platform NLCs in gel. (**A**) Strain (γ) sweep for the Carbopol^®^ gel alone, Carbopol^®^ gel containing Blank-NLCs and Carbopol^®^ gel containing CUR-NLCs. (**B**) Flow curves: viscosity (ɳ) in function of shear rate$\;(\dot{\gamma }$) for Carbopol^®^ gel alone, Carbopol^®^ gel containing Blank-NLCs and Carbopol^®^ gel containing CUR-NLCs. (**C**) Evaluation of time-dependent flow behavior (recovery test) for Carbopol^®^ gel alone, Carbopol^®^ gel containing Blank-NLCs and Carbopol^®^ gel containing CUR-NLCs.

As shown in Figure 13A, there were no statistically significant differences in the flow points of the gel alone or the gel containing NLCs (CUR or blank). In all cases, three regions could be distinguished in the graphs. At the beginning of the test, while G′ > G″ and their magnitudes remain constant, a consistent structure characteristic of the tridimensional network of a gel is observed. Thus, the addition of NLCs did not hinder the formation of the superstructure of the gel during the preparation. Then, while G′ > G″ is maintained but G″ increases and G′ decreases (roughly before γ = 2%), the gel superstructure starts to break until the crossover (G′ = G″). After this point, the viscous behavior prevails over the elastic behavior and there is no more a tridimensional structure. The formulation becomes fluid. Figure 13B indicates that the inclusion of NLCs in the formulation did not change the shear-thinning behavior of the gels. Even if for every sample viscosity decreases with an increasing shear rate, the gels rapidly recover their structure after undergoing a high-shear period of time, as depicted in Figure 13C. In the context of a topical application, this can be translated into easy squeezing from a tube, successfully staying in the skin, and finally, a good spreading during application.

### 3.6. CUR Penetration from CUR-NLCs and CUR-NLCs/Gel

Penetration studies were performed on synthetic Strat-M^®^ membranes. As described in Section 2.12, thanks to their multi-layer composition, this material is known to represent an intact skin barrier and is considered as a pertinent model in mimicking skin properties [38,39]. The timepoints assessed were 15 min, 30 min, 1 h and 24 h at pH = 5.5. The experimental temperature was set to 32 °C in order to simulate real-life topical application conditions.

**Figure 14:**
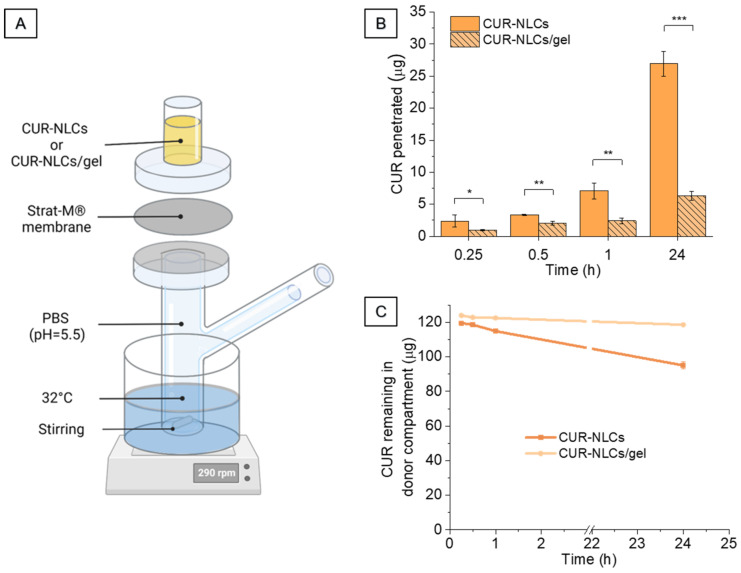
CUR penetration studies. (**A**) Experimental set-up of the Franz cell. (**B**) Penetration profiles of CUR into Strat-M^®^ membranes from CUR-NLC suspensions and CUR-NLCs incorporated into gel. (C) Amount of CUR remaining in the donor compartment at the end of each timepoint. Asterisks denote statistically significant differences. Two-sample Student’s *t*-test was carried out and statistically significant differences were identified when *p*-values were lower than 0.05 (* *p* < 0.05), 0.01 (** *p* < 0.01) or 0.001 (*** *p* < 0.001). (Created with BioRender.com (accessed on 26 April 2022)).

Figure 14B shows the CUR penetration profiles for CUR-NLCs and CUR-NLCs/gel. For the formulations, the initial CUR concentration in the donor compartment of the Franz cell was 122 ± 4 μg/mL and 125 ± 4 μg/g, respectively. CUR penetration in Strat-M^®^ membranes was time-dependent for both CUR-NLCs and CUR-NLCs/gel, but higher for CUR-NLCs at all timepoints. CUR penetration expressed as % from the initial amount in the donor compartment was 1.97 ± 0.76% and 0.78 ± 0.06% for CUR-NLCs and CUR-NLCs/gel after 15 min, respectively, and 22.06 ± 1.59% and 5.07 ± 0.53% for CUR-NLCs and CUR-NLCs/gel at 24 h, respectively. Table 5 shows the biopharmaceutical parameters of the amount of CUR entrapped per unit area (De) and the CUR accumulation (Ac) at 15 min and 24 h for CUR-NLCs and CUR-NLCs/gel.

Neither CUR nor NLCs were able to permeate across the membranes. DLS measurements of receptor compartment samples over 24 h (data not shown) presented the same count rate as PBS, indicating that NLCs were retained by the membrane. It was not possible to quantify CUR in the receptor compartment by either HPLC or spectrophotometry; however, Figure 14C shows that the decrease in the amount of CUR in the donor compartment corresponds to the amount of CUR penetrated into the membrane (Figure 14B), demonstrating that CUR was not able to cross the membranes.

## 4. Discussion

The combination of different carriers allows the attainment of advanced formulations. In this way, we proposed a biocompatible dual system composed of nanostructured lipid carriers (NLCs) and a Carbopol^®^-based hydrogel to locally deliver CUR in a controlled way while preserving its antioxidant properties.

NLCs were prepared by the hot homogenization method as described in previous works [35]. The lipids selected for NLC formulations are approved for pharmaceutical and/or cosmetic applications [42]. Precirol^®^ ATO 5 (Glyceryl palmitostearate) was chosen as solid lipid (3% *w*/*w*) because of its relatively low melting point (~55 °C), which allows minimal exposure of CUR to heat while forming a homogeneous lipid melt. Labrafac^®^ lipophile WL 1349 (Caprylic/capric triglyceride), also known as medium chain triglyceride (MTC), was used as liquid lipid (0.6 % *w*/*w*). As previously shown, the use of saturated lipids contributes to heat stability and limits the possibility of degradation [36]. Poloxamer 407 (3% *w*/*w*) and Tween^®^ 80 (0.4% *w*/*w*) were used as surfactants in aqueous and lipid phases, respectively. The amounts used for preparing NLCs are in the range of those found in the literature for topical formulations (10–25% for Precirol^®^ ATO 5 [43], 9% for Labrafac^®^ lipophile WL 1349 (caprylic/capric triglyceride), 1.0–8.4% for Tween^®^ 80 (https://www.cir-safety.org (accessed on 26 April 2022)) and 0.3–20% for Poloxamer 407) [44]. In these conditions, two populations of different sized negatively charged NLCs (P1, 70–90 nm and P2, 300–350 nm) were obtained. When results were expressed as % of number, P1 (70–90 nm) was the principal NLC peak found. Similar values have been reported by Tupal et al. (60–80 nm for mean size distribution and ~−11 mV for mean ZP) for NLCs containing similar lipid and surfactant compositions [45]. In addition, Singh et al. indicated a correlation between components of NLCs and their morphology, reporting a similar ovoid platelet shape and thickness values as our results [46]. CUR was successfully loaded into the optimized NLCs (DL% = 2.3%; EE% around 85%). These values are in accordance with those found in the-literature (85–95% of EE% and around 3% of DL%) for CUR-NLCs [47,48,49,50]. A purification step was introduced to eliminate the “not particulate material” (low molecular weight substances or aggregates), which could interfere with the performance of the biological assays. After SEC column purification, CUR quantification in NLCs showed that the loaded CUR and EE% was halved. CUR concentrations in the prepared NLC aqueous suspensions before and after the SEC column were found to be higher (170-fold and 84-fold, respectively) than the solubility of CUR in water (0.005 mg/mL) reported by Chambure et al. An increase of the apparent water solubility of CUR in NLCs has already been reported by the same authors [31]. This could be partially explained by the selected lipid matrix, which has been shown to provide an environment favoring CUR incorporation into the NLCs and be capable of maximizing CUR solubility [51,52,53]. In addition, components (lipids and surfactants) and amounts used for NLC preparation are reported to stabilize nanoparticles and prevent them from agglomeration [54]. In particular, the use of non-ionic surfactants as poloxamer 407 and Tween^®^ 80 has been associated with the formation of stable formulations through a steric repulsion phenomenon [55]. In this case, surfactant molecules present at the surface of nanoparticles could hinder the coalescence between them. This stability is supported by the homogeneous appearance of NLCs during the 45 days of observation after preparation.

In addition, our results show that NLCs preserve and improve the antioxidant activity of CUR (7-fold more after purification of the NLCs). This antioxidant activity enhancement by using NLCs has already been demonstrated in our previous work for other antioxidants (1.5-fold for astaxanthin-NLCs and 3-fold for supramolecular solvents-astaxanthin-NLCs) [33,36]. Concerning CUR, results are in accordance with those obtained by Ak and Gülçin [56], who tested the scavenging activity of various compounds, including CUR and α-tocopherol by different methods and showed the same tendency.

The effect of CUR and CUR-NLCs on the viability and metabolism was evaluated in two representative skin cells, fibroblasts and keratinocytes. Culture media containing CUR induced a decrease of viability and metabolic activity of both cell types at concentrations higher than 5 μM. CUR-NLCs shown no toxic effects for concentrations up to 10 μM for fibroblasts or keratinocytes. These results are in concordance with those described by Kloesch et al. [57], which showed a decrease in the toxicity of curcumin formulated in liposomes towards synovial fibroblasts and macrophages compared with non-encapsulated CUR. Furthermore, results suggested that the CUR-NLCs could preserve the metabolic activity of fibroblasts under stress conditions.

Additionally, in our work we observed that the treatment of both types of cells with CUR-NLCs leads to a reduction in their migration/proliferation ability after 24 h and to the keratinocytes morphology changes when compared with controls. In the same way, studies of CUR loaded on poly (lactic-co-glycolic acid)-nanoparticles showed strong inhibition of HaCaT human keratinocyte cell line activity [20]. Studies on mechanisms involved on the inhibition activity of CUR suggested a targeted action on the potassium channels of immune cells, which have a central role in chronic immune pathologies such as psoriasis [58]. For this reason, it has been suggested that CUR may be considered anti-psoriatic due to these anti-inflammatory effects [18,20].

All of the rheological results presented in this study may be explained by the nature of the interactions between NLCs and the microstructure of the Carbopol^®^ gel. It could be hypothesized that the formulated NLCs are free to move together within the Carbopol^®^ microparticles, which can reach diameters of 200 µm after swelling and neutralization. In consequence, the measured viscosity is that of the surrounding media. By tracking fluorescent particle trajectories, Oppong et al. [59] suggested that when this happens two regions coexist. A region formed by the highly cross-linked cores of the microgels, and a more viscous region containing the polymer chains. If particles are found in the first region, they are trapped and will have limited movement. Taken with the experiments performed in this study, under flow conditions it is probable that they would interfere with the behavior of Carbopol^®^ microgels. However, if they are found in the viscous region, the low density of entanglement in the polymer chains can form mesh sizes that will allow the particles to freely move. Thus, under flow conditions, the movement of the Carbopol^®^ microgels are not hindered by the particles. Kowalczyk et al. [60] showed that an increase in the Carbopol^®^ polymer concentration from 0.1% to 0.75% translated into a restriction in the movement of polystyrene particles of 510 nm diameter. This was due to the increased entanglement density at the viscous zones, which were responsible for diminishing the mesh size to below 500 nm. This might imply that the formulated NLCs could interfere with the flow behavior of the gel if higher Carbopol^®^ concentrations are used.

In healthy skin (and Strat-M^®^ membranes), the stratum corneum (or lipid coating for membranes) is the first barrier that NLCs would face in order to completely permeate. The fact that no NLC permeation was detected (thus, no risk of transdermal or systemic action in a real context) could be explained by their strong lipid nature, and thus strong affinity with the upper lipophilic layers in the Strat-M^®^ membrane [61,62]. Our results are in line with those of Rapalli et al. [30], who did not detect CUR permeation after 24 h of contact of CUR-NLCs with goat ear skin. They demonstrated that CUR was preferentially accumulated in the stratum corneum rather than in the viable epidermis and dermis. Some other studies have concluded that NLCs could enhance CUR permeation through animal skin samples with reported flux between 0.092 and 2.45 µg/cm^2^/h. However, composition of the receptor media containing at least 20% *v/v* of EtOH [63,64] can change thinking of the possibility of disruption of the skin barrier function (by lipid disordering or lipid extraction) [65,66]. In any of these studies, identification of NLCs in the receptor compartment was performed. Thus, the curcumin detected could also be the result of the NLCs dissolution by EtOH and subsequent passage to the receptor chamber through the disordered/disintegrated lipid barrier of the skin. In both, CUR-NLCs and CUR-NLCs/gel, the increase of CUR penetration in the Strat-M^®^ membrane with time could be explained by the ability of both formulations to increase the contact time with the membrane, which is important to have an effective transfer of NLCs. Lower CUR accumulation for CUR-NLCs/gel points to a decrease of NLC mobility due to the increase in the viscosity of the dispersant phase. This seems to allow fewer CUR-NLCs to come into contact with the membrane, and thus, a decrease in their transfer into it.

## 5. Conclusions

In summary of this work, we designed and developed an innovative dual NLCs/hydrogel system able to deliver a natural bioactive compound of high interest, CUR. Antioxidant activity of CUR was preserved and enhanced when entrapped into the NLCs. Moreover, the non-cytotoxic CUR-NLCs presented a moderate anti-migration/proliferation effect onto dermal cell lines and allowed CUR penetration into a Strat-M^®^ membrane. These results provide the proof of concept for considering this dual CUR delivery system as a potential candidate for specific skin applications and paves the way to offering new solutions to resolve challenging questions in existing critical dermal situations.

## Figures and Tables

**Table 1:** CUR content of NLCs before SEC column step.

	Direct Method	Indirect Method
Loaded CUR (mg/mL) ^1^	0.85 ± 0.04	0.83 ± 0.03
%EE ^2^	84.56 ± 4.48	83.40 ± 3.63
%DL ^3^	2.33 ± 0.10	2.23 ± 0.12

^1^ Loaded-CUR in NLCs refers to the mass concentration of CUR loaded in NLCs after preparation. ^2^ Encapsulation efficiency (%EE) of CUR-NLCs refers to the concentration of CUR incorporated into NLCs over the initial CUR concentration (1 mg/mL). ^3^ Drug loading (%DL) of CUR-NLCs refers to the amount of CUR incorporated into NLCs per weight of lipids (*w*/*w*). All values were obtained by direct and indirect method measurements. CUR determination in each batch was performed in triplicate (*n* = 3).

**Table 2:** CUR content of NLCs before and after SEC column obtained by direct method.

	Before SEC Column	After SEC Column
Loaded CUR (mg/mL) ^1^	0.85 ± 0.04	0.44 ± 0.04
%EE ^2^	84.56 ± 4.48	54.79 ± 6.09

^1^ Loaded-CUR in NLCs refers to the mass concentration of CUR loaded in NLCs. ^2^ Encapsulation efficiency (%EE) of CUR-NLCs refers to the mass concentration of CUR in fractions 3–5 after SEC (mg/mL), over CUR mass concentration before SEC (mg/mL). CUR determination in each batch was performed in triplicate (*n* = 3).

**Table 3:** Sizes (expressed as % of intensity) of CUR– and Blank-NLCs at 1 and 7 days before and after SEC column.

		Before SEC Column				After SEC Column			
Day		Blank-NLCs		CUR-NLCs		Blank-NLCs		CUR-NLCs	
		P1	P2	P1	P2	P1	P2	P1	P2
1	Size (nm)	77 ± 8	313 ± 37	72 ± 11	308 ± 36	89 ± 1	350 ± 16	72 ± 18	310 ± 24
7	Size (nm)	77 ± 10	318 ± 38	76 ± 15	362 ± 84	73 ± 23	329 ± 30	79 ± 18	348 ± 6

Results were done by duplicate (*n* = 2).

**Table 4:** Antioxidant activity measured by lipophilic ABTS assay.

	α−TEAC ^1^
CUR	0.47
CUR-NLCs before SEC	1.90
CUR-NLCs after SEC	3.31

^1^ α−TEAC is expressed as µM CUR/µM α-tocopherol. Each sample was analyzed in triplicate.

**Table 5:** Biopharmaceutical parameters for CUR and CUR-NLCs/gel.

Time (h)	Sample	Q_T_ (µg)	De (µg/cm^2^)	Ac (cm)
0.25	CUR-NLCs	2.40 ± 0.92	0.94 ± 0.36	0.0077 ± 0.0030
	CUR-NLCs/gel	0.97 ± 0.07	0.38 ± 0.03	0.0031 ± 0.0002
24	CUR-NLCs	26.92 ± 1.94	10.58 ± 0.76	0.0867 ± 0.0062
	CUR-NLCs/gel	6.33 ± 0.66	2.49 ± 0.26	0.0199 ± 0.0021

(Q_T_) represents the amount of CUR penetrated into membranes. (De) represents the amount of CUR entrapped per unit area, the area being available for penetration = 2.54 cm^2^. (Ac) represents the CUR accumulation.

## Data Availability

Not applicable.
